# Phase-transfer induced room temperature ferromagnetic behavior in 1T@2H-MoSe_2_ nanosheets

**DOI:** 10.1038/srep45307

**Published:** 2017-03-28

**Authors:** Baorui Xia, Tongtong Wang, Wen Xiao, Rongfang Zhang, Peitao Liu, Jun Ding, Daqiang Gao, Desheng Xue

**Affiliations:** 1 Key Laboratory for Magnetism and Magnetic Materials of MOE, Key Laboratory of Special Function Materials and Structure Design, Ministry of Education, Lanzhou University, Lanzhou 730000, P. R. China; 2 Department of Materials Science and Engineering, National University of Singapore, 117574, Singapore

## Abstract

Manipulating electronic and magnetic properties of two-dimensional transitional-metal dichalcogenides has raised a lot of attention recently. Herein we report the synthesis and ferromagnetic properties of phase-transfer induced room temperature ferromagnetic behavior in 1 T@2H-MoSe_2_ nanosheets. Experimental results indicate the saturated magnetization of the 1 T@2H-MoSe_2_ compound increases first and then decreases as the increasing of 1 T-MoSe_2_ phase, where 65.58% 1 T-MoSe_2_ phase incorporation in 2H-MoSe_2_ could enhance the saturated magnetization from 0.32 memu/g to 8.36 memu/g. Besides, obvious magnetoresistance behaviors are observed in these samples, revealing their potential applications in future spintronics.

Diluted magnetic semiconductors (DMS) have been of great interest due to their possibility for high temperatures beyond room temperature predicted by Dietl *et al*. in 2001^1^, where clear room temperature ferromagnetism (RTF) has been widely studied in oxide based diluted magnetic semiconductors such as ZnO and SnO_2_^2^,^3^,^4^,^5^. On the other hand, owing to the increasing need of micro devices in modern electronic, nano-scaled semiconductors are more and more popular in applying to these fields^6^,^7^,^8^. In this case, two-dimensional (2D) transitional metal dichalcogenides (TMDs) became the ideal materials to meet the demand for applications in nano-scaled semiconductor devices^9^,^10^,^11^. In recent years, TMDs have been investigated by many researchers owing to their excellent properties in various fields such as nanoscale field-effect transistors, phototransistors, sensors, lithium-ion battery, and photocatalysts^12^,^13^,^14^,^15^. Among these TMDs, MoX_2_ (X = S, Se) are the most studied materials, which are popular for their unique graphene-like structures (X-Mo-X), bounded together by van der Waals interactions^16^,^17^. Compared with graphene, MoX_2_ possesses intrinsic large band gaps (1.3–1.8 eV) in their monolayer form and flexibility of MoX_2_ atomic layers, which makes it possible for their applications in nanoelectronic and optoelectronic devices on both conventional and flexible substrates^18^,^19^,^20^,^21^. Beyond that, the well defined spin-splitting property of MoX_2_ makes them as promising spintronics devices^22^,^23^. However, with the development of spintronics, semiconductors possessing excellent magnetic properties are in great demand for applications^24^. Therefore, realizing and manipulating ferromagnetism in MoX_2_ nanosheets become the critical issue and challenging problems to be solved. Just like traditional DMSs materials, doping magnetic ions into MoS_2_ is an efficient way to induce the RTF, which are reported both theoretically and experimentally in recent years^25^. Results indicate that all the dopants induced ferromagnetism are mainly focusing on the defects, however, magnetic clusters, and secondary phases are possible as the main contributors to the observed ferromagnetism^26^,^27^,^28^. For this reason, some non-magnetic ions are selected as the dopants in experiments. As reported in our previous work, copper ions as the dopants are induced in MoS_2_ nanosheets to make it become ferromagnetic and obtained a high Curie temperature up to 930 K^29^. What’s more, many other ways to introduce ferromagnetism into two dimension TMDs materials have been explored both theoretically and experimentally. It is predicted that ferromagnetism appears when MoS_2_ nanoribbons are formed with zig-zag edges^30^,^31^,^32^,^33^,^34^. Besides, the intrinsic structure transformation could be an efficient method to introduce robust RTF^35^,^36^, where the structure transformation related RTF in MoSe_2_ is seldom reported. In this paper, we synthesize 1 T@2H-MoSe_2_ nanosheets with different phase rations by two-step solvothermal method. Results indicate that the 1 T@2H-MoSe_2_ nanosheets with different phase rations show the variation magnetic properties. where 65.58% 1 T-MoSe_2_ phase incorporation in 2H-MoSe_2_ could enhance the saturated magnetization from 0.32 memu/g to 8.36 memu/g. Besides, the obvious magnetoresistance behaviors reveal their intrinsic RTF and their potential applications in future spintronics.

## Results and Discussion

**Figure 1:**
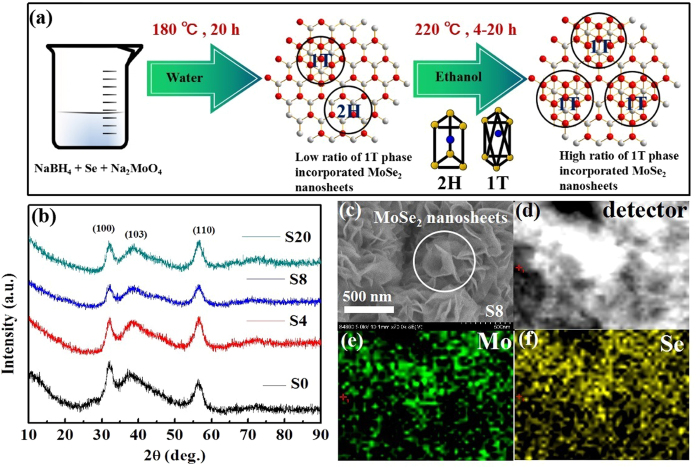
Experimental details and basic structure of MoSe_2_. (**a**) Two-step of solvothermal method for preparing MoSe_2_ nanosheets, in which the solutions were selected as distilled water and ethanol. (**b**) X-ray diffraction (XRD) patterns of S0, S4, S8 and S20. (**c**) Scanning electric microscope (SEM) image of pristine MoSe_2_ nanosheets. (**d**–**f**) EDS-mapping images of S8: (**d**) detector, (**e**) molybdenum and (**f**) selenium.

We synthesize 1 T@2H-MoSe_2_ nanosheets by two-step solvothermal method (the schematic diagram is shown in Fig. 1(a))^35^ where phase-transferred MoSe_2_ nanosheets with 0 h, 4 h, 8 h, and 20 h are labeled as S0, S4, S8 and S20 respectively in the following passage. X-ray diffraction (XRD) patterns of the samples are shown in Fig. 1(b). According to the standard PDF card of MoSe_2_ (JCPDS No. 29–0914), the diffraction peaks are located at 31.4°, 37.9°, and 55.9°, which are related to certain MoSe_2_ crystal planes of (100), (103), and (110), respectively. The 2H or 1 T structure can not only be identified by these three peaks owing to the further atomic structure should be investigated, the results of which will be discussed in what follows in the passage. Figure 1(c) shows the SEM image of synthesized MoSe_2_ nanosheets (S8), and it can be seen that the obtained MoSe_2_ nanosheets are condensed and assembled thin layers. SEM images of other samples are provided in Supplementary Information. Besides, EDS analysis results of S8 are presented in Fig. 1(d–f). Within the uniform distribution of light and shade contrast of elemental images captured by a detector, the existence of molybdenum and selenium can be easily observed, demonstrating the element consistence of MoSe_2_ nanosheets.

**Figure 2:**
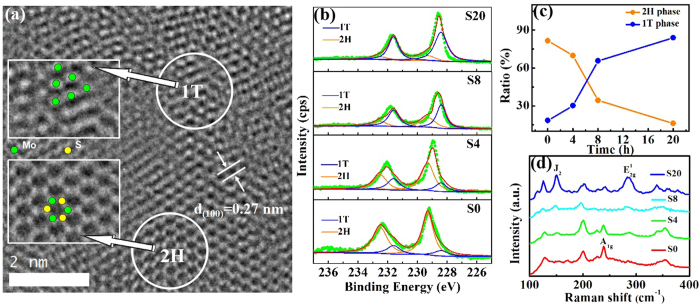
Characterized for 1 T incorporated 2H structures of MoSe_2_ nanosheets. (**a**) High resolution transmission electron microscope (HRTEM) image of S8, the two insets of which are enlarged images of 2H and 1 T structure regions. (**b**) X-ray photon spectra (XPS) results of Mo 3*d* core level in S0, S4, S8 and S20. (**c**) 1 T and 2H concentrations of four synthesized samples plotted according to the XPS results. (**d**) Raman spectra of four samples.

Figure 2(a) is the 1 T@2H TEM image of S8, two different regions are obviously compatible. We can see typical molybdenum atoms in either 2H or 1 T structure possess six selenium atoms, which are triangular prism and octahedral configuration, respectively (white circles). Additionally, the panels can be seen in high resolution transmission electron microscope (HRTEM) image and the interplanar spacing is calculated to be 0.27 nm, indicating the (110) panels of 2H MoSe_2_ (JCPDS No. 29-0914). Figure 2(b) shows the high resolution X-ray photoelectron spectra (XPS) of the four samples. In terms of Mo 3*d* regions, all the spectra can be well fitted by two sets of peaks. The peaks around 229.3 eV and 232.5 eV correspond to 3*d* 5/2 and 3*d* 3/2 components of 2H structure MoSe_2_. Yet once the 1 T structure is induced, these two peaks will shift to lower binding energies of 228.4 eV and 231.6 eV. As shown in Fig. 2(c), the 1 T concentrations of S4, S8 and S20 are calculated from Mo 3*d* spectra as 30.19%, 65.58% and 83.95%, respectively. Figure 2(d) shows the Raman spectra of these four samples, from which the peaks at 150.7 cm^−1^ and 289.4 cm^−1^ in S4, S8 and S20 can be observed. These two peaks, marked as J_2_ and E_2g_^1^, are the exclusive peaks of MoSe_2_ 1 T structure, consisting with the work reported by Uttam *et al*.^37^. Herein, the intensity of these two peaks growing with the amount of 1 T phase, with respect to A_1g_ peak, which also demonstrates the phase transformation from 2H to 1 T.

**Figure 3:**
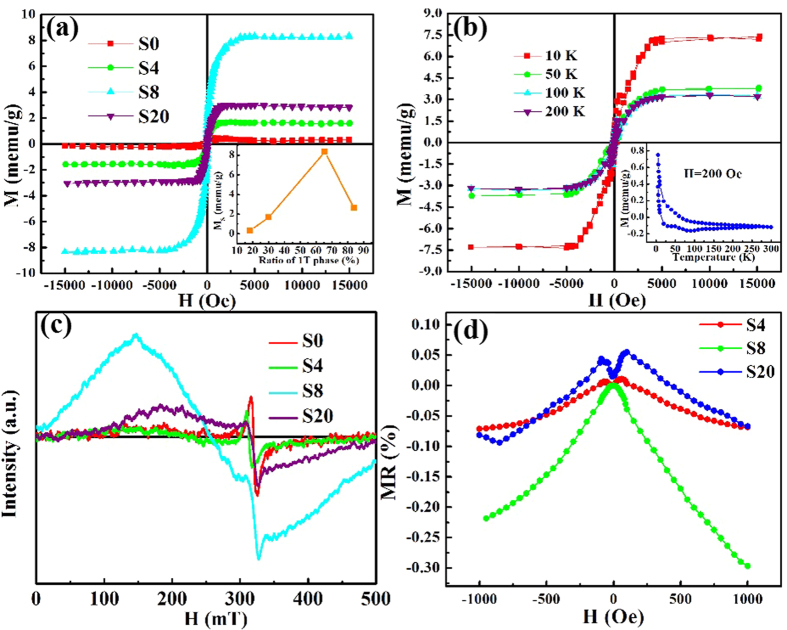
Magnetism properties of 1 T incorporated 2H MoSe_2_ nanosheets. (**a**) M-H hysteresis loops of S0, S4, S8 and S20, inset is the saturate magnetization with respect of 1 T structure concentration in MoSe_2_ nanosheets. (**b**) M-T loops of S4 in varies temperatures: 10 K, 50 K, 100 K and 200 K. The inset shows the zero-field cooled (ZFC) and field cooled (FC) curves of S4. (**c**) Electron spin resonance (ESR) patterns of S0, S4, S8 and S20. (**d**) Magnetoresistance (MR) of S4, S8 and S20.

Besides, we investigate the magnetic properties of 1 T@2H-MoSe_2_ nanosheets. The room temperature magnetic hysteresis loops of samples are shown in Fig. 3(a), where the linear background signals have been subtracted^38^. Compared with S0, the saturate magnetization (M_s_) for S4 and S8 increases from 0.32 memu/g to 1.6 memu/g, and then to 8.36 memu/g, respectively, suggesting that introduction of 1 T phase could lead to the ferromagnetic ordering in MoSe_2_ nanosheetes. From the inset of Fig. 3(a), it can be seen that the M_s_ increases until the 1 T concentration raises to 65.58%, dramatically, the M_s_ decreases to 2.6 memu/g in S20 (83.95% 1 T phase). Figure 3(b) gives the isothermal hysteresis loops of S4 from 10 K to 300 K, the inset of which shows the zero-field-cooled (ZFC) and field-cooled (FC) curves. Typical ferromagnetism property has been characterized by these curves, thus the M_s_ decreases with the increasing of the measured temperature. The ZFC-FC curves suggest that the Curie temperature of the sample is above the room temperature. Besides, no blocking temperature can be found during the cooling process, indicating that there is no ferromagnetic cluster occurs in S4^39^. Besides, the electron spin resonance spectra (ESR) of four 1 T@2H-MoSe_2_ samples are shown in Fig. 3(d). As we can see, the resonance occurs in 325 mT (g = 1.98), corresponding to the paramagnetic resonance of the four samples. Besides, the distinct resonance signal raises up in S4, S8 and S20, nearly at 250 mT (g = 2.57), corresponding to the ferromagnetic resonance of these MoSe_2_ nanosheets, in accord with the M-H results in Fig. 3(a). In addition, obvious magetoresistance (MR) behaviors are observed in sample S4, S8 and S20. As described in Fig. 3(d), MR values are negative with the magnetic field range of [−0.1 T, 0.1 T] and evolves from 0% to −0.22% with the magnetic field increased to 0.1 T. While for the S4 and S20, the lowest MR value are only −0.07% and −0.09%, respectively. The MR values vary with the saturate magnetization of three samples, also confirm the observed ferromagnetism is intrinsic in 1 T phase incorporated MoSe_2_ nanosheets^40^.

**Figure 4:**
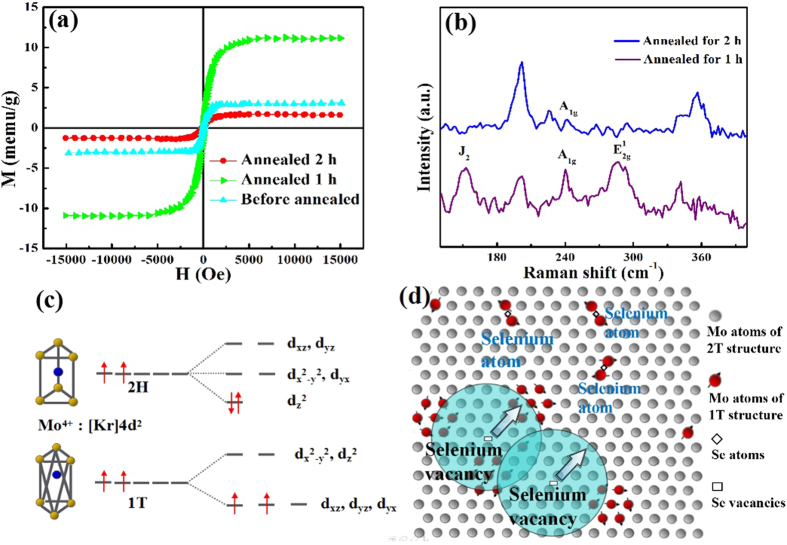
Discussions for origin of ferromagnetism in MoSe_2_ nanosheets. (**a**) M-H loops of S20 and annealed for 1 h and 2 h. (**b**) Raman spectra of S20 annealed for 1 h and 2 h. (**c**) The occupation of electrons in Mo 4*d* orbits under the crystal fields of 1 T phase and 2H phase. (**d**) Schematic diagram of BMPs in 1 T@2H MoSe_2_, where the red balls represent the Mo atoms of 1 T structure, and the gray balls represent the Mo atoms of 2H structure.

Above results indicate that the M_s_ decreases when the concentration of 1 T phase increases up to 83.95%. Therefore, we assume that the observed ferromagnetism is related to the relative ratio of both 1 T and 2H phase in MoSe_2_ nanosheets. To verify this, it is necessary to conduct further experiment. Zhao *et al*. report that transformation from 1 T to 2H can be conducted by annealing the 1 T samples under Ar ambitions^41^, so we perform the re-transformation with annealing the S20 in high purity Ar for 1 h and 2 h under 250 °C, respectively. The obtained samples are subsequently studied by using M-H hysteresis loops and Raman spectra. It can be seen from Fig. 4(a), the M_s_ increases by four times after annealing for 1 h, but decreases as the annealing time prolonged to 2 h. In Fig. 4(b), the peak of A_1g_ mode appears together with the peaks of J_2_ and E_2g_^1^ modes decaying after annealed for one and two hours. Both the two results indicate that the coexistence of 1 T and 2H phase is the ultimate condition for observed ferromagnetism in MoSe_2_ nanosheets.

To explore the origin of the observed ferromagnetism, it is necessary to point out how the magnet moment induced with the 1 T phase incorporated in 2H matrix. As we all know, 4*d* orbital has five degenerate states, called as *d*_*xz*_, *d*_*yz*_, *d*_*xy*_, *d*_*x*_^*2*^_*-y*_^*2*^ and *d*_*z*_^*2*^. In 2H phase, hexagonal symmetry configuration could induce splitting of 4*d* orbitals into three orbitals of closely spaced energies. In this case, these five orbitals unit into three groups: *d*_*xz*_ *& d*_*yz*_, *d*_*x*_^*2*^_*-y*_^*2*^ *& d*_*xy*_ and *d*_*z*_^*2*^, as described in Fig. 4(c), two 4*d* electrons of Mo^4+^ occupied *d*_*z*_^*2*^ orbital spin-antiparallely, as a result of which the Mo atoms in 2H phase structures exhibit nonmagnetic. While for 1 T phase of the MoSe_2_, the Mo atoms are surrounded by six Se atoms with octahedral coordination, therefore, the five orbits unit into two groups: *d*_*xz*_ *& d*_*yz*_ *& d*_*xy*_ and *d*_*z*_^*2*^ *& d*_*x*_^*2*^_*-y*_^*2*^. The *d*_*xz*_ *& d*_*yz*_ *& d*_*xy*_ orbits have lower energy level, therefore, determined by Hund’s rule, the two 4*d* electrons occupied solely in two of them spin-parallelly, causing the net magnet moments of 1 T phase Mo atoms^35^,^37^. For the robust ferromagnetism observed in 1 T@2H MoSe_2_ nanosheets, bounded magnetic polaron (BMP) model is suitable to explain the magnetic origin. Figure 4(d) gives the schematic diagram of BMPs in 1 T incorporated MoSe_2_ nanosheets. During the solvothermal synthesis process, many selenium vacancies formed in MoSe_2_ nanosheets, as a result, BMPs could be developed with localized holes and a large number of Mo^4+^ spins are bounded around the Se vacancies. The Mo^4+^ spins near a Se vacancies could align their spins parallel to the vacancy spin, leading to the formation of a BMP. It has been reported by Cai *et al*. that the 2H-1 T transformation always occurs near the defects^35^. Based on this phenomenon, we proposed that in low 1 T concentration case, the Mo^4+^ spins emerged near the selenium vacancies and form BMPs. These BMPs began to overlap and ferromagnetic coupled, giving the origin of ferromagnetism and risen of magnetization. However, when the amount of 1 T concentration added up to 83.95%, the 1 T regions expand and produce more Mo^4+^ spins in the regions where the Se vacancy density is much lower, as shown in Fig. 4(d). In this case, majority of these Mo atoms are around Se atoms compared with Se vacancies, and they are either anti-ferromagnetic coupled by Se atoms or existed as isolated Mo^4+^ spins, resulting the decreasing of magnetization macroscopically. This could cause the weaken of ferromagnetism in MoSe_2_ nanosheets and this is why we observe the decreased M_s_ in S20.

## Conclusions

In summary, we synthesize the 1 T phase incorporated 2H-MoSe_2_ nanosheets by solvothermal method, the crystallinity of all samples have been confirmed by structural characterization methods. After the phase transformation, RTF of MoSe_2_ can be improved, with the M_s_ from 0.32 memu/g up to 8.36 memu/g. At the same time, the obtained MoSe_2_ nanosheets exhibit obvious magnetoresistance behavior with MR value up to −0.22% when the external magnetic field applied to ±0.1 T. The induced Se vacancies may affect the formation of the BMPs and their interactions, in turn controlling the magnetic moments of the 1 T phase incorporated MoSe_2_ nanosheets. The obtained results enlighten on the development of ferromagnetic MoSe_2_ nanosheets and provide them a paradigm of application of spintronics devices.

## Additional Information

**How to cite this article**: Xia, B. *et al*. Phase-transfer induced room temperature ferromagnetic behavior in 1T@2H-MoSe_2_ nanosheets. *Sci. Rep.* **7**, 45307; doi: 10.1038/srep45307 (2017).

**Publisher's note:** Springer Nature remains neutral with regard to jurisdictional claims in published maps and institutional affiliations.

## Supplementary Material

Supplementary Information
