# Supplementary material for: Phase-transfer induced room temperature ferromagnetic behavior in 1T@2H-MoSe2 nanosheets
Source: Sci Rep. 2017 Mar 28;7:45307. doi: 10.1038/srep45307 (PMC5368601; doi:10.1038/srep45307)
Supplement: Supplementary Information [file srep45307-s1.doc]

**Supplementary Information for Phase-transfer induced room temperature ferromagnetic behavior in 1T@2H-MoSe_2_ nanosheets**

Baorui Xia^1^, Tongtong Wang^1^, Wen Xiao^2^, Rongfang Zhang^2^, Peitao Liu^1^, Jun Ding ^2^, Daqiang Gao1,2[[1]](#footnote-2)*, Desheng Xue^1*^.

^1^ Key Laboratory for Magnetism and Magnetic Materials of MOE, Key Laboratory of Special Function Materials and Structure Design, Ministry of Education, Lanzhou University, Lanzhou 730000, P. R. China.

^2^ Department of Materials Science and Engineering, National University of Singapore, 117574, Singapore

**S1. Experimental details**

We synthesized 1T@2H-MoSe_2_ nanosheets by two-step solvothermal method. In first step, 0.304 g of NaBH_4_ (≥96.0 %), together with 0.316 g (0.004 mol) Se powder (≥99.9 %) were dissolved in 75 ml distilled water and stirred for 30 min upon a magnetic stirrer. Subsequently, 0.484 g (0.002mol) Na_2_MoO_4_ (≥99 %) was added in the solution and continued stirring for 5 min. Then the mixture was transferred into a 100ml steel autoclave and heated at 180 ℃ for 20 h. After that, powders obtained from the first step were rinsed for several times and transferred in an 100 ml steel autoclave. To our presuming, the content of 1T phase in MoSe_2_ nanosheets is positively correlate with the reaction time, therefore, we heated the autoclaves at 220 ℃ for 4 h, 8 h, and 20 h, marked as S4, S8 and S20 respectively, as shown in figure 1(a). For convenience of comparison, we had also synthesized pristine MoSe_2_ nanosheets without the second step treatment, marked as S0.

X-ray diffraction spectra (XRD, Philips X’Pert) were recorded by using a system with Cu Kα radiation (wave length=0.154 nm). Transmission electron microscopy (TEM, Tecnai TMG2F30, FEI) was carried out to investigate the microstructure of the samples. The morphologies of the MoSe_2_ nanosheets were characterized by using a scanning electron microscope (SEM; Hitachi S-4800). Raman spectra (Jobin-Yvon HR 800 spectrometer) was also recorded, the wavelength of laser light used in our experiment is 532 nm and the exposure time is 15 s. X-ray photoelectron spectra (XPS, Kratos AXIS Ultra^DLD^) of MoSe_2_ nanosheets were measured to analyzing the elements of MoSe_2_ nanosheets. The hysteresis curves were measured on a vibrating sample magnetometer (VSM Model EV9, MicroSense, LLC). The hysteresis curves measured at lower temperature by using a super quantum interfere device (SQUID, Quantum Design). Additionally, we used an I-V multimeter (Keithley2400) with an external magnetic field measuring magnetoresistance of samples.

**S2. Morphology of all the samples**

The SEM images of S0, S4, S8 and S20 are shown in Fig. S1. It can be seen that all the nanosheets exhibit similar morphology and there is no significant difference among them.


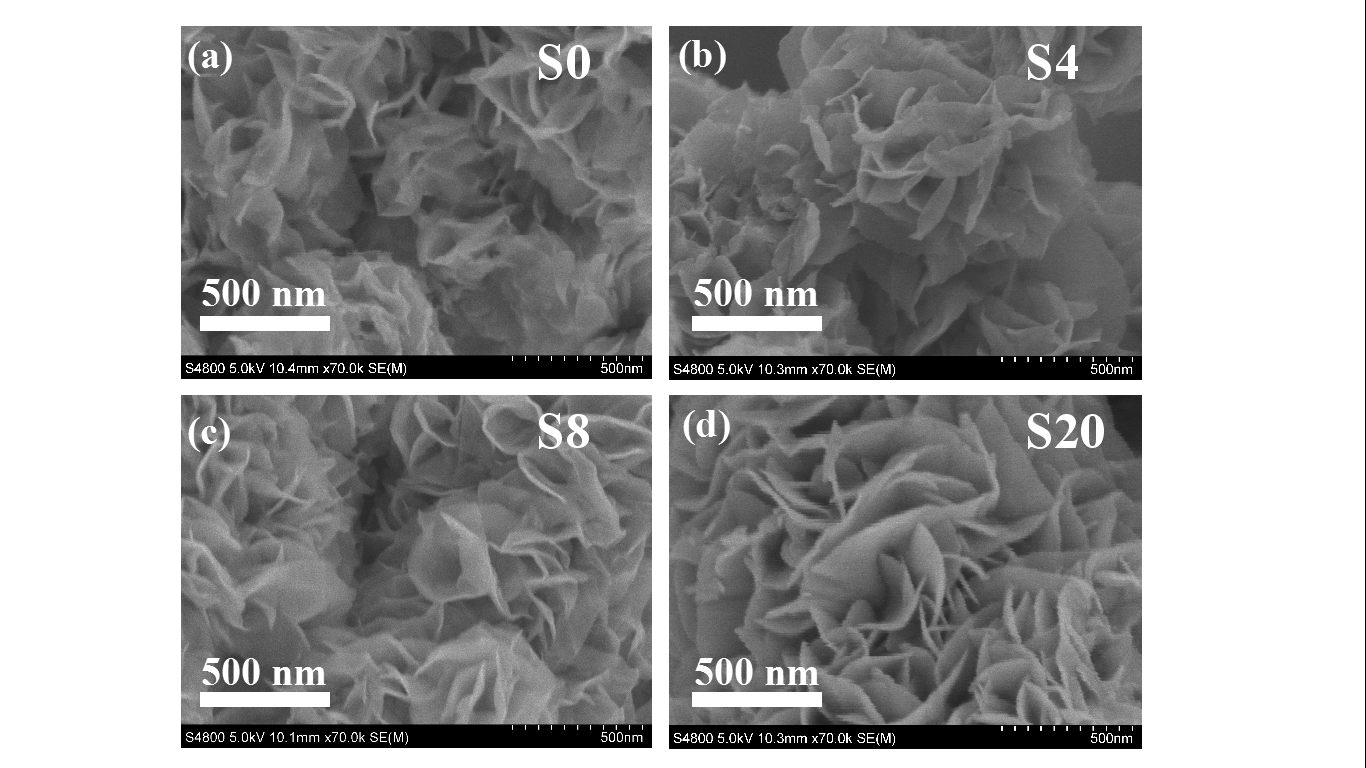


Fig. S1 SEM images of S0, S4, S8 and S20.

**S3. Magnetoresistance measurement**

To investigate the magnetoresistance (MR) properties, the samples were pressed as thin slices for measurement. We selected powders of S4, S8 and S20 and put them into moulds respectively. All these samples were pressed in a jack under the pressure of 20 MPa for 15 min. Subsequently, the obtained slices were fixed on PEB substrates, every slice was sticked two copper wires for using as electrodes.

1. ^*^ Corresponding author: *E-mail address*: [gaodq@lzu.edu.cn;](mailto:gaodq@lzu.edu.cn;) [xueds@lzu.edu.cn](mailto:xueds@lzu.edu.cn) [↑](#footnote-ref-2)
